# A Nonradioactive Fluorimetric SPE-Based Ceramide Kinase Assay Using NBD-C_6_-Ceramide

**DOI:** 10.1155/2012/404513

**Published:** 2012-07-26

**Authors:** Helena Van Overloop, Gerd Van der Hoeven, Paul P. Van Veldhoven

**Affiliations:** Department Cellular and Molecular Medicine, Katholieke Universiteit Leuven, Campus Gasthuisberg O&N1, LIPIT, Herestraat, Box 601, 3000 Leuven, Belgium

## Abstract

Ceramide kinase (CERK) has been implicated in important cellular processes such as inflammation and apoptosis. Its activity is usually measured using radiolabeled ceramide or [**γ**-^32^P]-ATP, followed by extraction, thin-layer chromatography, and detection of the formed labeled ceramide-1-phosphate. To eliminate the use of radioactivity, we developed similarly but independently from the approach by Don and Rosen (2008), a fluorescence-based ceramide kinase assay, using N-[7-(4-nitrobenz-2-oxa-1,3-diazole)]-6-aminohexanoyl-sphingenine (NBD-C_6_-ceramide) as substrate. Its *K*_*m*_ value (4 **μ**M) was comparable to that of N-hexanoyl-sphingenine (C_6_-ceramide). The produced fluorescent NBD-C_6_-ceramide-1-phosphate was captured by means of solid-phase extraction on an aminopropyl phase, resulting in a fast and sensitive CERK measurement. By performing this assay in a 96-well format, it is also suitable for high-throughput screening (HTS) to search for CERK modulators. A limited screen revealed that some protein kinase inhibitors (e.g., U-0126; IC_50_ 4 **μ**M) and ceramide analogues (e.g., fenretinide, AMG-9810; IC_50_ 1.1 **μ**M) affect CERK *in vitro*.

## 1. Introduction

Ceramide, the initial product of the sphingomyelin cycle, functions as a key component in the regulation of various cellular functions like differentiation, proliferation, apoptosis, and inflammation [1, 2]. In the past years, ceramide-1-phosphate (Cer-1-P), a metabolite of ceramide and sphingomyelin, also gained more attention and turned out to be a powerful bioactive sphingolipid. Cer-1-P has been implicated as a regulator of different cellular processes, like mitosis, apoptosis, phagocytosis, and inflammation [3, 4]. In mice lacking CERK, neutrophil homeostasis is defective leading to more severe disease upon pulmonary infection [5]. Until now, CERK is the only mammalian enzyme known to phosphorylate ceramide, but the residual Cer-1-P levels in tissues of the CERK-deficient mice [5], indicate the existence of alternative pathways to generate Cer-1-P. CERK was first described in 1989 as a Ca^2+^-dependent lipid kinase [6] and cloned in 2002 by Sugiura et al. [7], based on similarity to sphingosine kinase. A similar cloning strategy was followed by others [8]. CERK appears to associate with (endo)membranes via a pleckstrin domain [9, 10] and is highly selective for the D-*erythro* configuration of ceramides [8, 11].

**Figure 1:**
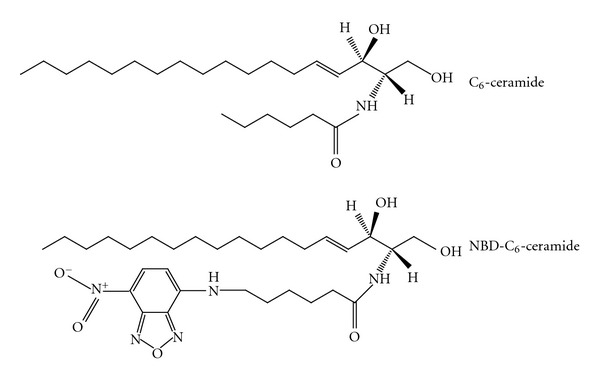
Structure of C_6_-ceramide and NBD-C_6_-ceramide.

CERK activity is commonly determined using a radioactivity-based assay [7–11] based on [*γ*-^32^P]ATP. After extraction of lipids, the amount of radiolabeled ceramide-1-phosphate in the organic phase is determined by directly counting [12] or by TLC followed by autoradiography and quantitation [7, 8]. To avoid the use of radioactivity, we intended to develop a fluorescence-based CERK assay, sensitive enough to be employed for cellular work and whose format would be suitable or could be adapted to search for CERK inhibitors via HTS. Based on previous work, showing that truncated ceramides such as C_2_-ceramide (N-acetyl-sphingenine) and C_6_-ceramide (N-hexanoyl-sphingenine), when presented bound to albumin, are well recognized by human CERK [8, 13], we tested a fluorescent analogue, namely, N-[7-(4-nitrobenz-2-oxa-1,3-diazole)]-6-aminohexanoyl-sphingenine (NBD-C_6_-ceramide, NBD-C_6_-Cer), in which NBD is coupled to sphingenine via an 6-aminohexanoic acid linker (see Figure 1). This lipid was introduced several years ago by Pagano and coworkers for the study of sphingolipid metabolism and shown to be metabolized in a similar way as ceramide, being incorporated into NBD-sphingomyelin and NBD-cerebrosides [14]. During these and subsequent studies its phosphorylation, as far as we are aware of, was never detected or described, likely due to the very low activity of CERK compared to the other ceramide utilizing pathways. Also when using ceramides containing a shorter N-acyl chain (truncated ceramides), phosphorylation by intact cells is difficult to reveal, requiring ^32^P-uploading of the cells as reported for neutrophils [15, 16], cerebellar granule cells, [17] and Hela cells [18]. Upon overexpression of CERK, detection of intracellular formed truncated ceramide-1-^32^P is facilitated [8, 13].

Here, we studied the kinetics, revealing that NBD-C_6_-Cer is a good substrate for CERK, both *in vitro* and *in vivo*, and developed a simple solid phase extraction scheme to measure CERK activity (this work was presented in a preliminary form at the LKI Oncoforum meeting, February 15, 2008, Leuven (Belgium) (H. Van Overloop and P. P. Van Veldhoven, Development of HTS-assays for enzymes acting on bioactive sphingolipids, new players in chemotherapy resistance. Part I. ceramide kinase)). At the start of this work, Graf et al. [19] reported that NBD-C_6_-Cer is phosphorylated when given to CERK-expressing Cos-1 cells, as revealed by TLC of cellular extracts and scanning. Independently, Don and Rosen [20] have described in the meantime a CERK assay using the same substrate but based on liquid/liquid extraction, phase separation, and transfer of the upper phase for analysis.

## 2. Materials and Methods

### 2.1. Expression of Lipid Kinases

Recombinant *Hs*CERK was expressed in Top10F' *E. coli *cells transformed with plasmid pPVV072, coding for a (His)_6_-tagged fusion of *Hs*CERK, as described before [8]. The harvested bacteria, resuspended in PBS (25 mL/100 mL culture) containing a mix of protease inhibitors, were sonicated on ice (Branson Sonifier B115, microtip), followed by a clearing step (10,000 g for 10 min). Aliquots of the supernatant were frozen in liquid nitrogen, stored at −80°C and diluted 1/15 in PBS containing protease inhibitors before use. Compared to the pelleted fraction [8], the specific activity of the soluble lysate fraction is three fold lower (9.3 nmol/min·mg protein at 100 *μ*M C_6_-Cer/40 *μ*M BSA for the batch used in these experiments), but it was considered to more compatible with SPE work up.

Recombinant human sphingosine kinase 1 (*Hs*SphK1) was obtained from Top10F' cells transformed with plasmid pSG003 as described before [21].

### 2.2. Synthesis of NBD-Hexanoyl Derivatives

 N-[7-(4-nitrobenz-2-oxa-1,3-diazole)]-6-aminohexanoic acid (33 *μ*mol, Molecular Probes), dissolved in 3 mL anhydrous dimethylformamide and activated with carbonyldiimidazole (40 *μ*mol, Fluka), was mixed with *D,erythro*-sphingenine (33 *μ*mol; Acros Organics), dissolved in 2 mL dimethylformamide, and stirred overnight at room temperature. After drying the reaction mixture, the amides were phase-separated in chloroform/methanol/water (1/1/0.9), the lower phase was dried, dissolved in 0.5 mL 33% methylamine in ethanol/water (7/3), and heated to 70°C for 90 min [22] to remove any formed O-acylated products. After evaporating the hydrolysis mixture, NBD-C_6_-Cer was extracted and further purified by preparative TLC (silica G60; Merck) in solvent A (chloroform/methanol/acetic acid, 93/7/1, v/v). Stock solutions were standardized by nitrogen determination (yield 63%) and purity, based on fluoroscanning (Storm 840 with blue LED (450 nm), GE Healthcare) after TLC separation in solvent B (chloroform/acetone/methanol/acetic acid/water, 10/4/3/2/1, v/v) was 89%, substantially better than that of commercially obtained NBD-C_6_-Cer (Sigma; Avanti Polar Lipids).

 NBD-C_6_-ceramide-1-phosphate (NBD-C_6_-Cer-1-P) was prepared by phosphorylation of homemade NBD-Cer using bacterially expressed *Hs*CERK under the conditions described before [8]. Briefly, the reaction mixture containing 100 *μ*M NBD-C_6_-Cer, solubilized with ethanol/BSA, was incubated with excess recombinant *Hs*CERK for 1 h at 37°C. After acidic phase separation, the lower phase was dried and the phosphorylated NBD-C_6_-Cer was further purified by preparative TLC (silica G60; Merck) in solvent B, followed by elution with chloroform/methanol/water (5/5/1, v/v). Stock solutions were standardized by organic phosphate content (yield 85%). Based on fluoroscanning of the phosphate ester, TLC-separated in solvent B, purity was estimated at >82% (based on main spot; due some streaking, actual purity is higher), being slightly better compared to the phosphate ester prepared from commercially obtained NBD-C_6_-ceramide (Avanti Polar Lipids, >75%).

### 2.3. Separation of NBD-C_6_-Cer and NBD-C_6_-Cer-1-P via Solid-Phase Extraction (SPE)

 To document the separation of NBD-labeled CERK substrate and product, 100 *μ*L CERK assay mixture containing NBD-C_6_-Cer and NBD-C_6_-Cer-1-P (both at 5 *μ*M final concentration) was mixed with 300 *μ*L methanol and applied on a 25 mg NH_2_-SPE column (Varian), which had been conditioned with methanol and water. SPE devices were washed and bound lipids were eluted as described further. Total fluorescence of the flow-through and eluted fractions was measured by fluorimetry (*λ*_ex_ 465 nm; *λ*_em_ 535 nm; Tecan Infinite 200). The amount of NBD-C_6_-Cer and NBD-C_6_-Cer-1-P in the different fractions was estimated by drying them, redissolving the residu in chloroform/methanol (1/1, v/v), followed by separation on silica G TLC (solvent B), and scanning of the fluorescent spots.

### 2.4. Ceramide Kinase Measurements

 To prepare the reaction mixture for the fluorescent CERK assay, largely based on previous work [8], NBD-C_6_-Cer was dissolved in ethanol and mixed with 4 volumes of BSA (resulting in a molar ceramide/BSA ratio of 2.5), followed by addition of reaction mixture up to 75 *μ*L and a 25 *μ*L aliquot of recombinant CERK or cell lysate. Final concentrations were 5 *μ*M NBD-C_6_-Cer−1 mM ATP−50 mM Mops/NaOH pH 7.2−3 mM MgCl_2_−40 mM NaF−1 mM dithriothreitol−100 *μ*M orthovanadate. After 10 min at 37°C, the reaction was stopped by addition of 300 *μ*L methanol. The mixture was applied to an NH_2_-column (25 mg, Chromabond Multi-96, Macherey-Nagel), which had been activated with 0.5 mL methanol followed by 0.5 mL water. After washing the column with 800 *μ*L methanol containing 2% formic acid, followed by 100 *μ*L methanol containing 0.5 M trifluoroacetic acid (TFA), NBD-C_6_-Cer-1-P was eluted into a black FIA 96-well plate (Greiner) with 250 *μ*L methanol containing 3 M TFA. Fluorescence was measured in a multi-reader (*λ*_ex_ 465 nm, *λ*_em_ 535 nm; Tecan Infinite 200). The amount of NBD-C_6_-Cer-1-P in the eluate was calculated based on a calibration curve with NBD-C_6_-Cer-1-P at concentrations of 0 to 5 *μ*M treated simultaneously and equivalently with the samples. Fluorescence measurements from calibration curves fitted to a linear equation (*R* ^2^ = 0.998; *n* = 12).

CERK activity based on radioactivity was measured as described before [8], but using 1 mM [*γ*-^32^P]-ATP (GE Healthcare) and reducing the volumes to obtain similar conditions as in the fluorescent assay described above. As substrates C_6_-ceramide (40 *μ*M) or NBD-C_6_-Cer (5 *μ*M), both bound to BSA (molar ceramide/BSA ratio = 2.5), were used.

## 3. Results and Discussion

**Figure 2:**
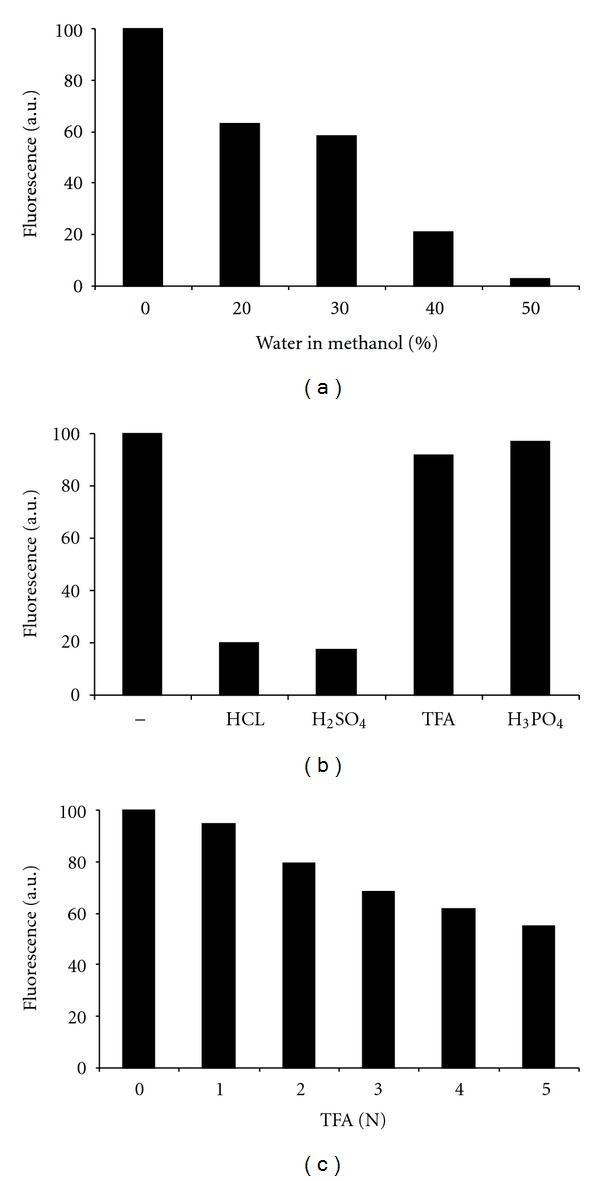
Influence of water and acidity on fluorescence of NBD-derivatives. Fluorescence of methanolic NBD-C_6_-Cer-1-P solutions (1 *μ*M, 200 *μ*L) containing increasing amounts of water (a) different acids at 0.5 N final concentration (b) or increasing TFA concentration (c) (*λ*_ex_ 465 nm, *λ*_em_ 535 nm; Tecan Infinite 200).

 The substrate specificity of CERK, documented by different groups [7, 8, 11], reveals that the N-acyl chain can be shortened up to two carbons [7, 8, 13] and that the presence of a bulky group in this chain is tolerated (unpublished data). Ceramide analogues with a shortened base are also phosphorylated [8]. Previously, we documented that natural ceramides (with a long N-acyl chain) are better recognized by CERK when presented in a micellar form, whereas less hydrophobic ceramide analogues, either with a shortened base or a truncated acyl chain, display better activity in the presence of BSA [8, 13]. In agreement with this observation, NBD-C_6_-Cer, containing the polar NBD moiety, was well phosphorylated when bound to BSA, but substantially less when incorporated in octylglucoside/cardiolipin micelles, certainly at low substrate concentration (30 fold, less at 25 *μ*M). Similarly, use of Triton X-100 [11] or CHAPS micelles resulted in low activities (data not shown). Using bacterially expressed human CERK in a radiometric assay and separation of the products by TLC, a *K*_*m*_ of 6 *μ*M was obtained for NBD-C_6_-Cer, bound to BSA (data not shown). Despite the bulky NBD-group, this value is about 2.5-fold lower than the *K*_*m*_ for the nonfluorescent C_6_-ceramide (14 *μ*M), obtained under the same assay conditions. A similar *K*_*m*_, but based on fluorimetry, was obtained by Rosen and Don (1 *μ*M in presence of 7 *μ*M BSA), who also documented the inhibitory action of Triton X-100 [20]. The *K*_*m*_ for ATP was 168 *μ*M (Figure 2(b)).

Given the low *K*_*m*_, it appeared justified to evaluate some procedures to separate NBD-C_6_-Cer from NBD-C_6_-Cer-1-P in order to develop a nonradioactive CERK assay. In addition, we attempted to avoid tedious liquid extraction steps and aimed for a procedure compatible with HTS. Hence, to separate the fluorescent substrate and product, after some trials on reversed phase (NBD-C_6_-Cer and its phosphate ester were both retained on C18-SPE (Varian) at 50% methanol, without or with addition of acid (0.5% (v/v) TFA) or base (0.5% (v/v) triethylamine), but coeluted with pure methanol; similar findings were obtained with Oasis-HLB (Waters) columns, except that NBD-C_6_-Cer-1-P was recovered in the flow through under alkaline conditions.) and ion exchange-SPE, we focused finally on NH_2_-SPE columns. These systems have been employed to separate phospholipids, and lipids containing a primary phosphate group, such as phosphatidate and phosphorylated phosphoinositides, do bind strongly [16, 17]. Elution of these phosphate esters is achieved by increasing the polarity of the eluting solvent and addition of strong acids such as phosphoric acid [23] or HCl [24].

Given the reported dependence of the fluorescence of NBD-derivatives with regard to the lipid environment [25], solvent composition, polarity and pH [25–27] and low quantum yield in water [25], in a first step the fluorescence of NBD-C_6_-Cer and its phosphate ester was evaluated in solvents and in the presence of acids. For solvent we focused on the use of methanol, being suitable for dissolution of these lipids, compatible with plastic (e.g., polystyrene), and having a low toxicity and moderate cost. As shown in Figure 2(a), increasing the amount of water in a methanolic NBD-C_6_-Cer-1-P solution, strongly reduced the fluorescence. Compared to pure methanol as solvent, fluorescence dropped to 3% in a solution containing 50% of water. The influence of different acids, present at 0.5 N final concentration, on the fluorescence of NBD-C_6_-Cer-1-P, dissolved in methanol, is shown in Figure 2(b). Both HCl and H_2_SO_4_ caused a severe drop in fluorescence (less than 20% compared to the neutral methanolic solution). The influence of TFA and H_3_PO_4_ was less drastic, since more than 90% of fluorescent signal remained (Figure 2(b)). This effect seems to be related to the strength of the acid, although it is not strictly linearly related to the acid dissociation constant.

When applied in pure methanol or methanol containing up to 25% water, NBD-C_6_-Cer-1-P was quantitatively retained on NH_2_-SPE systems. Both TFA and H_3_PO_4_ displaced NBD-C_6_-Cer-1-P from the NH_2_-phase, but TFA was chosen for further optimisation. Less volume was required to elute the phosphate ester compared to methanol containing H_3_PO_4_ in equal normality, and the decrease in NBD fluorescence at increasing TFA concentrations was rather small (Figure 2(c)), both factors improving the sensitivity of the assay. By increasing the TFA concentration to 3 N, it was possible to elute the bound lipid in a small volume, 250 *μ*L when using 25 mg SPE.

**Figure 3:**
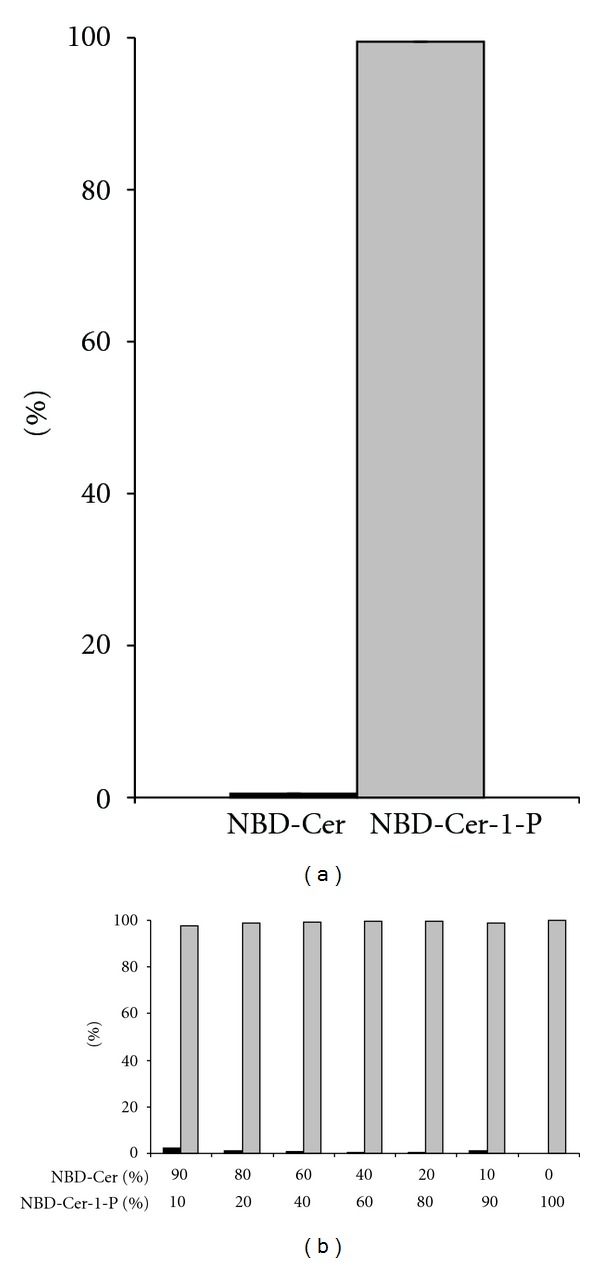
Separation of NBD-C_6_-Cer and NBD-C_6_-Cer-1-P via NH_2_-SPE. A mixture containing NBD-C_6_-Cer and NBD-C_6_-Cer-1-P, both at 5 *μ*M (a) or at a total concentration of 5 *μ*M but with a variable ratio (b) in the assay medium was separated via NH_2_-SPE, as described in Section 2. The eluted fractions were dried, resolubilized in chloroform/methanol (1/2, v/v) and the lipids were separated on silica G TLC plates (chloroform/acetone/methanol/acetic acid/water, 10/4/3/2/1, v/v), followed by fluorescence scanning of the spots (NBD-C_6_-Cer (black bars); NBD-C_6_-Cer-1-P (grey bars).) The result is expressed as percentage of total fluorescence in the elution fraction (a) mean ± SEM; *n* = 5; (b) single experiment).

Finally, we analyzed how well NBD-C_6_-Cer and its phosphate ester could be separated using small SPE systems (25 mg NH_2_-SPE, column format or contained in a 96-well format). Hereto, a mixture containing NBD-C_6_-Cer and NBD-C_6_-Cer-1-P and with a similar composition as the CERK assay mixture, was diluted with methanol (75% final concentration), and applied to the NH_2_-SPE column, which had been conditioned with methanol and water. After washing the column with methanol containing 2% formic acid (FA) (800 *μ*L) and 0.5 N TFA (100 *μ*L), NBD-C_6_-Cer-1-P was eluted from the column using methanol containing 3 N TFA (250 *μ*L). This resulted in a good separation between NBD-C_6_-Cer and NBD-C_6_-Cer-1-P (If employing a larger SPE format, adjust volumes accordingly; e.g. for 100 mg NH_2_-SPE columns, NBD-C_6_-Cer-P is eluted with a similar yield with 1 ml 3 N TFA in methanol (data not shown)). When loading equal amounts of both lipids, more than 99% of NBD-C_6_-Cer was present in the flow-through and wash fractions (99.6 ± 0.01%, mean ± SEM, *n* = 5), and less than 0.4% was found in the acidic eluate (0.36 ± 0.02%), based upon fluorescence-scanning of the TLC-separated fractions (data not shown). Fluorescence in the TFA-eluate was almost completely (99.4 ± 0.08%) associated with NBD-C_6_-Cer-1-P (Figure 3(a)). Apparently, these numbers do not change when varying the relative amounts of both fluorescent lipids initially present (Figure 3(b)): about 96% of the total amount of NBD-C_6_-Cer-1-P is present in the eluted fraction (95.6 ± 0.42%). The use of TFA at lower normality than 3 N resulted in a lower recovery for NBD-C_6_-Cer-1-P (data not shown). Higher normalities did not improve the recoveries, but resulted in lower sensitivity because of increased quenching.

**Figure 4:**
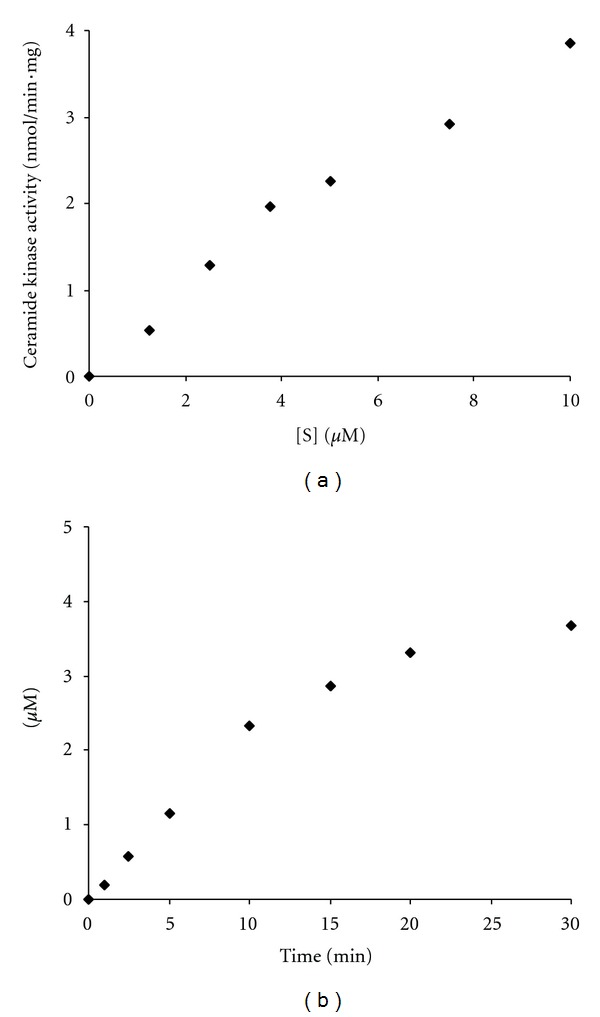
Substrate and time dependence of the kinase reaction. Recombinant bacterially expressed *Hs*CERK was incubated with the indicated concentration of NBD-C_6_-Cer (a) or 5 *μ*M (b) in the presence of 1 mM ATP at 37°C. The reaction was stopped at 10 min (a) or the indicated time periods (b) by addition of methanol and the mixture was applied to an NH_2_-SPE column. The reaction product NBD-C_6_-Cer-1-P was quantified based on fluorescence intensity of the column eluate and converted into nmol or *μ*M, based on a calibration curve.

Having established optimum SPE-separation conditions, the kinetics of CERK were reevaluated with the new assay. The assay conditions were similar to the traditional radiometric assay [8], but assay volume was reduced to 100 *μ*L and ATP concentration was fixed at 1 mM. In addition, soluble CERK was used to avoid potential SPE clogging. To halt the reaction, 3 volumes of methanol were added, followed by transfer of the mixture to the 96-well SPE-plate. A *K*_*m*_ value of 4 *μ*M was obtained (Figure 4(a)), comparable to the *K*_*m*_ of 6 *μ*M obtained for NBD-C_6_-Cer with the radiometric assay (see above). For economical use of the substrate, its concentration was fixed at 5 *μ*M (standard assay conditions). At this concentration, although close to the *K*_*m*_, phosphorylation continued at a linear rate till about 50% of the substrate was converted (Figure 4(b)). Likewise, when varying the amounts of CERK, production of NBD-C_6_-Cer-1-P proceeded linearly till about 50% of NBD- C_6_-Cer was phosphorylated (data not shown). Hence, the amount of NBD-C_6_-Cer converted under the standard conditions is a good measure for CERK activity.

**Figure 5:**
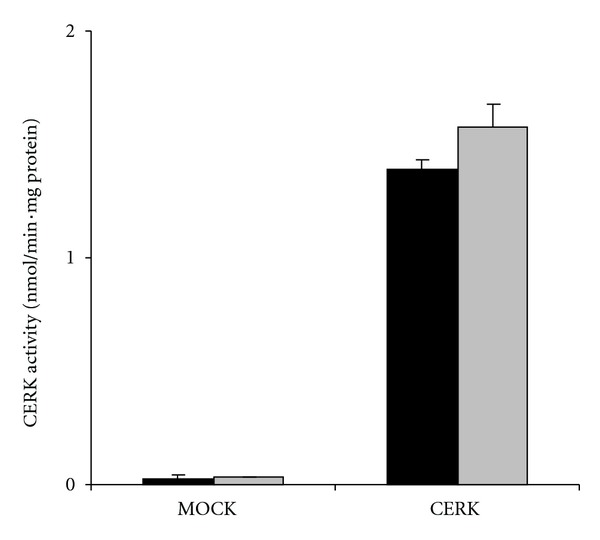
CERK activity in cultured cells. CERK activity towards NBD-C_6_-Cer was determined in lysates from CHO cells, transfected with pCMV-Tag2B (mock) or pHVO001, coding for a Flag-HsCERK fusion [8], using the fluorescence assay (black bars) or the radiometric assay (grey bars, mean ± SEM, *n* = 3). CERK activity is expressed as nmol per mg protein per min (nmol/min·mg protein).

As documented in Figure 5, the fluorescent assay is very convenient to document CERK activity in cultured cells. Upon overexpression of CERK, a ±50-fold increase in kinase activity (1.40 versus 0.029 nmol/min·mg protein) was measured. Similar values were obtained using the radiometric assay (1.57 versus 0.030 nmol/min·mg protein), supporting the use of the fluorescence assay as a valuable alternative. The detection limit of the assay is estimated at 10 pmol NBD-C_6_-Cer-1-P, meaning that CERK activities corresponding to 1 pmol/min can be measured (or less if incubation time is prolonged). When relying on a [*γ*-^32^P]-ATP based assay, and labelling of the produced ceramide-1-^32^P to 100–1000 dpm, this would require an input of 0.5–5 *μ*Ci/assay at 1 mM ATP and 100 *μ*L assay volume. Since CERK activity in most tissues and cells is quite low (<40 pmol/min·mg protein) [8], our assay will facilitate further work on CERK and its regulation. In addition, the low *K*_*m*_ implicates that NBD-C_6_-Cer might be a handy substrate for *in vivo* CERK measurements. Indeed, when added to CERK-expressing cultured cells, formation of NBD-C_6_-Cer-1-P can be followed by TLC analysis of the cellular lipid extracts (data not shown), in full agreement with data reported by Bornancin and coworkers [19]. By comparing the scanned intensities against the fluorescence of NBD-C_6_-Cer-1-P standards, TLC analysis of cell extracts is another means to calculate CERK activity (data not shown). During our attempts to publish this work, Don and Rosen [20] reported on the same ceramide analogue as a substrate but their assay was based on either TLC spotting for the micellar assay or for the BSA-based assay, extraction, followed by phase separation and transfer of the upper phase for analysis; the latter was done in a 96-well format. The solvent influence on the NBD-fluorescence was apparently not considered.

**Figure 6:**
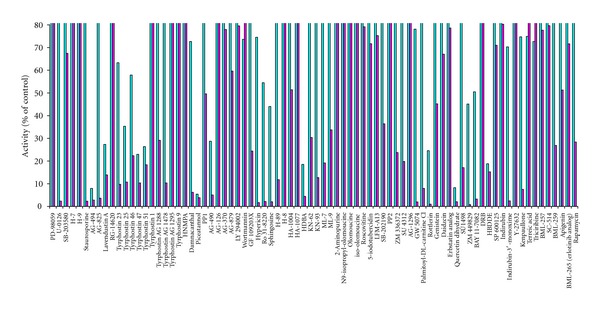
Influence of proteine kinase inhibitors on CERK via 96 well HTS. CERK was measured with NBD-C_6_-Cer in the presence of 0.5 mM of established protein kinase inhibitors (kinase inhibitor library, Biomol), using the 96 well fluorescent SPE assay. Activity is expressed as % of the control containing 10% DMSO (100 ± 11%; mean ± SD; *n* = 3, magenta bars). CERK was not influenced by DMSO, up to 20% (data not shown). For comparison, effect of the inhibitors on sphingosine kinase is displayed in blue bars as % of control (100 ± 7%; mean ± SD; *n* = 3).

Omission of a liquid-liquid extraction step clearly speeds up the assay and allows for other formats like multiwell plates used in HTS. To simulate an HTS, a commercial library was tested in a 96 well format. To increase the chance to get some positive hits, we selected hereto a protein kinase inhibitor library given that their targets rely on the same cofactor as CERK. To show specificity, the same library was also tested on another lipid kinase, human sphingosine kinase 1. Various established protein kinase inhibitors appear to affect CERK (Figure 6). CERK activity was blocked (more than 95% inhibition) by AG-494, AG-825, BAY11-7082, 2-hydroxy-5-(2,5-dihydroxybenzylamino)benzoic acid, hypericin, indirubin-3′-monoxime (and its 6-bromo-derivative), piceatannol, quercetin, Ro31-8220, rottlerin, *D-erythro*-sphingosine (and *D-threo*-sphinganine; not shown), U-0126, staurosporine, and ZM449829 (at 500 *μ*M). SPHK1 was clearly inhibited by fewer compounds, the most potent being AG-494, piceatannol, and quercetin. For the most potent CERK-inhibitors, IC_50_ values were determined: U-0126 (4 *μ*M), followed by 6-bromo-indirubin-3′-oxime (9 *μ*M) and hypericin and rottlerin (both 19 *μ*M). U-0126 is considered to be a selective MAP kinase kinase inhibitor and IC_50_ values are indeed lower (72 nM for MEK1; 58 nM for MEK2) [28].

A few ceramide analogues and lipophilic amides, partly commercially obtained, partly homemade, were also tested as substrate and/or inhibitor (full list available upon request). Compounds that were not phosphorylated but strong inhibitory were further evaluated. From this screen, we retained fenretinide {(N-4-hydroxyphenyl)retinamide); IC_50_ 1.1 *μ*M} en AMG-9810 {(E)-3-(4-t-butylphenyl)-N-(2,3-dihydrobenzo [b][1,4]dioxin-6-yl)acrylamide; IC_50_ 1.4 *μ*M}. These compounds are known to influence other biological processes. Fenretinide binds f.i. the retinoic acid receptor, slows the growth of transformed cells, and induces apoptosis in cultured cells (effective concentrations 1–10 *μ*M) [29], the latter likely via increasing dihydroceramide levels [30]. AMG-9810 is known as an antagonist of the vanilloid/TPRV1 receptor [31]; its endogenous ligand, anandamide, is also a fatty amide.

Summarizing, by further analyzing the substrate spectrum of CERK, it was shown that NBD-C_6_-ceramide is a suitable substrate, allowing for a fluorescence based CERK measurement. By combining this substrate with the use of NH_2_-SPE to isolate the product, a straightforward assay has been developed, useful for basic research (100 mg SPE) and adaptable to HTS for CERK inhibitors/activators (25 mg SPE-96 well format). Recently, a HTS-CERK assay was described by Munagala et al. [32], which can be miniaturized to 1,536 well plates. However, this assay is based on chemiluminescent detection of the disappearing ATP and C_12_-ceramide as substrate. Hence, an extra control is required for the effect of compounds on the coupling reaction/enzymes. Moreover, this assay is not applicable to crude cell/tissue lysates given the interfering presence of ATPases and other phosphatase activities [33] and the low CERK activity.

## Abbreviations

C6-: Hexanoyl-
Cer-1-P: Ceramide-1-phosphate
CERK: Ceramide kinase
HTS: High throughput screening
NBD: 7-nitrobenz-2-oxa-1,3-diazole
SPE: Solid phase extraction
NBD-C-Cer: N-[NBD]-6-aminohexanoyl-sphingenine or NBD-C-ceramide
NBD-C6-Cer-1-P: N-[NBD]-6-aminohexanoyl-sphingenine-1-phosphate or NBD-C-ceramide-1-phosphate
TFA: Trifluoroacetic acid.
